# Pollen Food Allergy Syndrome in Japanese Children and Adolescents: Risk Factors and Pollen Sensitisation

**DOI:** 10.1155/2023/4075264

**Published:** 2023-03-09

**Authors:** Masaya Kato, Manabu Miyamoto, Fumitaka Takayanagi, Yusuke Ando, Yuji Fujita, Motoko Nakayama, Shigemi Yoshihara

**Affiliations:** Department of Pediatrics, Dokkyo Medical University, Tochigi, Japan

## Abstract

Pollen food allergy syndrome (PFAS) is caused by cross-reactivity with pollen; however, not all-pollen-sensitised individuals develop PFAS, and studies on the characteristics of PFAS development are limited in Japan. We investigated the prevalence and risk factors for the development of PFAS in Japanese children and adolescents sensitised to pollen and their association with pollen-specific IgE levels. The characteristics of PFAS were investigated in patients with allergies aged 3–18 years who visited Dokkyo Medical University Hospital between January 2016 and December 2019. Specific IgE levels for alder, Japanese cedar, ragweed, and orchard grass were measured in patients sensitised to any of the pollens. Patients were categorised into preschool (G1), elementary school (G2), and middle-high school (G3) groups. Overall, 600 patients were enrolled. The prevalence of PFAS was 8.5% in G1, 20% in G2, and 36.3% in G3. Multivariate logistic regression analysis demonstrated strong associations between the risk of developing PFAS and older age (odds ratio (OR), 1.12; 95% confidence interval (CI), 1.06–1.19; *P* < 0.001), seasonal allergy rhinitis (OR, 6.93; 95% CI, 1.59–30.34; *P* = 0.010), and alder sensitisation (OR, 6.20; 95% CI, 2.66–14.49; *P* < 0.001). Spearman's correlation revealed statistically significant positive correlation between each pollen-specific IgE level; high pollen-specific IgE levels were also a risk factor. The OR for being sensitised to all four species was 36.83 (95% CI, 8.93–151.83, *P* < 0.001) when compared with Japanese cedar alone. Alder was most relevant, with an alder-specific IgE level cutoff value of 2.54 UA/mL. The sensitivity was 78.9%, and the specificity was 70.9%. In conclusion, preschool children develop PFAS with alder sensitisation, and higher pollen-specific IgE levels and increased number of pollen sensitisations are risk factors for developing PFAS.

## 1. Introduction

Pollen food allergy syndrome (PFAS) is caused by cross-reactivity with pollen antigens and is classified as a Class 2 food allergy [1–3]. The major allergens of PFAS are Bet v 1 homologs, also known as pathogenesis-related protein type 10 (PR-10), and profilin proteins [4]. Because they are often digested by heat or enzymatic processing, PFAS symptoms, also known as oral allergy syndrome (OAS), are confined to the oral cavity [2]. Recently, it has been reported that cross-reactivity of pan-allergens, such as nonspecific lipid transfer protein, lipid transfer protein (TLP), and gibberellin-regulated protein (GRP), cause systemic symptoms [5–7]. Additionally, systemic symptoms caused by PR-10 and profilin have also been reported [8]. However, up to 3% of individuals with PFAS experience systemic symptoms without oral symptoms and 1.7% experience anaphylactic symptoms, with the majority experiencing OAS symptoms [2]. The only source of sensitisation for Bet v 1 homologs is birch tree pollen (birch and alder, among others) [9–11]. Alternatively, profilin sensitises a wide variety of pollens, from birch trees to grasses (e.g., orchard grass and timothy), and weeds of the Asteraceae family (e.g., ragweed and mugwort) [12, 13].

The prevalence of PFAS is known to vary by region due to the type of pollen and effect of the amount of pollen in the air. In children with allergic diseases, the reported prevalence is 4.7–48% [14–17]. The majority of seasonal allergic rhinitis in Japan is caused by Japanese cedar pollen [18]. The prevalence of Japanese cedar pollinosis between 1998 and 2019 increased significantly: 1.7–3.8% among 0–4-year-old individuals, 7.5–30.1% among 5–9-year-old individuals, and 19.7–49.5% among 10–19-year-old individuals [19]. Tomatoes have been identified as a food that is cross-reactive with cedar pollen [20] but have been reported only in few cases [21]. PFAS in Japan has also been associated with birch trees, grasses, and Asteraceae plants [17]. The prevalence of non-Japanese cedar pollinosis in 2019 was 2.6% among 0–4-year-old individuals, 17.4% among 5–9-year-old individuals, and 33.8% among 10–19-year-old individuals [19], with more than half of Japanese cedar pollinosis patients also suffering from other pollinosis. Thus, the incidence of PFAS in children and adolescents is expected to increase in Japan as pollinosis increases. However, few studies have investigated pollen and the development of PFAS in children and adolescents. Not all people sensitised to pollen develop PFAS, and few reports have examined the risk factors for developing PFAS in children and their association with pollen sensitisation and pollen-specific IgE levels. In this study, we investigated risk factors for the development of PFAS in sensitisers of Japanese cedar, alder, ragweed, and orchard grass (the main pollinators in Japan), in children and adolescents aged 3–18 years. Their pollen-specific IgE levels were also examined in relation to the risk factors for developing PFAS as well as whether pollen-specific IgE levels could predict onset of PFAS.

## 2. Materials and Methods

### 2.1. Study Design

This study is a single-centre retrospective study of medical records. Patients were not required to provide informed consent as this study used anonymous clinical data obtained after each patient had agreed to medical activities by written consent. We also applied the opt-out method to obtain informed consent by poster that was approved by the institutional review committee. This study was conducted in accordance with the ethical principles of the Declaration of Helsinki and Ethical Guidelines for Medical and Health Research Involving Human Subjects by the Ministry of Health, Labour and Welfare of Japan. This study was approved by the Ethical Review Board of Dokkyo Medical University (ID: R-7-21).

### 2.2. Study Participants

Participants were patients with allergies who were followed up at the Department of Pediatrics, Dokkyo Medical University Hospital in Tochigi prefecture (an area approximately 100 km north of Tokyo), from January 2017 to December 2019. The enrolment criteria were as follows: (i) patients aged 3–18 years, who were classified into preschool (G1: 3–6 years), elementary school (G2: 7–12 years), and middle-high school (G3: 13–18 years); (ii) diagnosis with one or more of seasonal allergic rhinitis (sAR), bronchial asthma (BA), food allergy (FA), and atopic dermatitis (AD); (iii) patients with serum-specific IgE measured for alder, Japanese cedar, ragweed, and orchard grass; and (iv) to be sensitised to any of the aforementioned allergens. The serum-specific IgE levels were measured using Immuno CAP (Thermo Fisher Scientific, Uppsala, Sweden). An allergen-specific IgE level of ≥0.35 UA/mL was regarded as positive.

The exclusion criteria were as follows: (i) patients receiving treatment or drugs that affect specific IgE production, such as immunotherapy, biological therapies, and oral steroids; (ii) any other severe chronic disease; and (iii) other conditions deemed unsuitable by the physician in charge.

### 2.3. Diagnosis of Allergic Diseases

The diagnoses of sAR, BA, FA, and AD were made by the allergy specialists in accordance with the Japanese guidelines [22–25]. AD included past history, while the other diseases were defined as current diagnosis. The diagnosis of sAR was defined as pollen sensitisation and the presence of three symptoms: sneezing and nasal itch, watery rhinorrhoea, and nasal blockage in pollen season.

### 2.4. Diagnosis of PFAS

The diagnosis of PFAS was made through questionnaires and clinical history. The questionnaire included the following questions: (i) Have you experienced any symptoms within 15 minutes after ingesting any of the foods listed below? (ii) If any, which symptoms have you had? The food list contained the major fruit and vegetable allergens in Japan: apple, pear, peach, cherry, watermelon, melon, banana, kiwi, orange, pineapple, strawberry, mango, grape, loquat, plum, carrot, avocado, eggplant, cucumber, tomato, and celery. The symptom category included oral allergy symptoms (tingling/itching sense of oedema of the lips, oral cavity, and/or throat) or systemic symptoms (itching or urticaria, cough, dyspnoea, vomiting, and/or anaphylaxis).

PFAS in this study was defined as oral allergy or systemic symptoms within 15 min after ingesting raw foods listed above. Fruit and vegetable allergies in children include Class 1 food allergies, and thus, those with only systemic symptoms were excluded from the PFAS group. Additionally, nut allergies were excluded owing to the large number of Class 1 food allergies in children. Allergy specialists took a clinical history using questionnaires to render a diagnosis of PFAS.

### 2.5. Statistical Analyses

For the analysis, serum-specific IgE levels below the lower limit of quantitation (<0.10 UA/mL) were assigned a value of 0.09 UA/mL for statistical calculations, while serum-specific IgE levels over the higher limit of quantitation (>100 UA/mL) were assigned a value of 101 UA/mL. Serum-specific IgE levels are normally distributed during logarithmic transformation; therefore, they were transformed to the ordinary logarithm for examination. Fisher's exact test, pairwise comparison (Fisher's exact test with Bonferroni correction) was used to compare data among the three groups by age. The chi-square test was used for the number of pollen sensitisations and incidence of PFAS. Spearman's rank correlation coefficients for each pollen-specific IgE level were also calculated. In multiple logistic regression analyses, odds ratios (ORs) were used to analyse the risk of developing of PFAS. Univariate logistic regression was used for pollen-specific IgE levels and the incidence of PFAS. Receiver operating characteristic (ROC) analysis and Youden's index were used to calculate cutoff values, sensitivity, and specificity for pollen sensitisation and PFAS. Statistical analyses were performed using SPSS, version 26 (IBM Corp., Armonk, NY, USA). Statistical significance was set at *P* < 0.05.

## 3. Results

### 3.1. Patients' Clinical Characteristics

A total of 600 patients were included in the study. The demographic characteristics of the patients are shown in Table 1. G1, G2, and G3 comprised 199, 244, and 157 children, respectively. The median age of patients at the time of blood sampling was 9 (range, 3–18) years, and 378 (63.0%) patients were male individuals. There were 123 (20.5%) patients with PFAS. Overall, 48.0% of patients had AD, 33.3% had BA, and 77.0% had sAR. Proportions of pollen-specific IgE positivity were 61.8%, 99.0%, 56.3%, and 63.3% for alder, Japanese cedar, ragweed, and orchard grass, respectively. The prevalence of PFAS was 8.5% in G1, 20% in G2, and 36.3% in G3; these were significantly different among the three groups (*P* < 0.001) and tended to increase with age. There were no sex differences in the prevalence of PFAS in any age group (G1: *P* = 0.296, G2: *P* = 0.243, and G3: *P* = 0.865). FAs excluding PFAS were significantly different among G1, G2, and G3 (*P* < 0.001) and tended to decrease with age. sAR was higher in G2 and G3 than in G1 (*P* < 0.001) and was not significantly different between G2 and G3 (*P* = 0.522). The sensitisation of alder, ragweed, and orchard grass was increased in G2 and G3 compared with that in G1 (*P* < 0.001) but did not differ between G2 and G3 (alder: *P* = 0.581, ragweed: *P* = 0.598, and orchard grass: *P* = 0.052). Japanese cedar was not significantly different among these groups (*P* = 0.099).

### 3.2. Causative Foods

The most common foods in the 123 cases of PFAS were peaches, apples, kiwis, melons, pineapples, strawberries, tomatoes, pears, watermelons, oranges, cherries, and bananas (Table 2).

### 3.3. Association between the Number of Pollen Sensitisations and Development of PFAS

**Figure 1:**
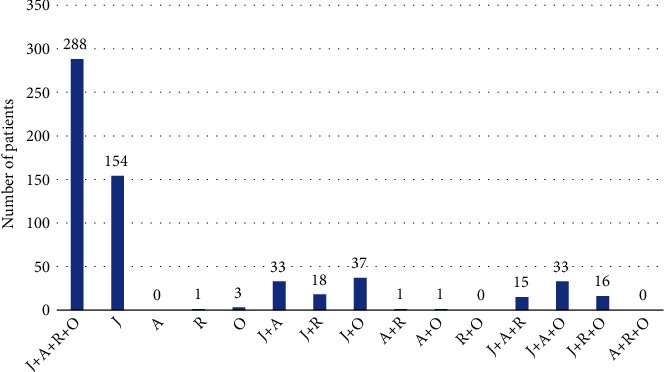
The number of patients by type of pollen sensitisation. J: Japanese cedar; A: alder; R: ragweed; O: orchard grass.

Overall, 288 (48%) patients were positive for four-pollen sensitisation (alder, Japanese cedar, ragweed, and orchard grass). Further, 154 (25.7%) patients were positive for Japanese cedar only and 90 (15.0%) were positive for two-pollen sensitisation. Moreover, 64 (10.7%) patients were positive for three-pollen sensitisation (Figure 1).

Two of 154 patients developed PFAS and were positive for Japanese cedar only; 13 of 90 were positive for two antigens; 14 of 64 were positive for three antigens; and 94 of 288 were positive for four antigens. The ORs were obtained from a chi-square test for patients positive for Japanese cedar only, two antigens (OR, 9.74; 95% CI, 2.08-45.57; *P* < 0.001), three antigens (OR, 21.28; 95% CI, 4.67–96.88; *P* < 0.001), and four antigens (OR, 36.83; 95% CI, 8.93–151.83; *P* < 0.001) (Table 3).

### 3.4. Correlation of Each Pollen-Specific IgE Level

**Figure 2:**
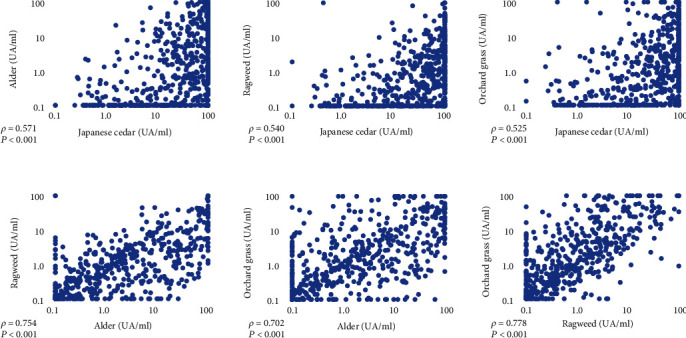
Scatter plot and Spearman's rank correlation coefficient of each pollen-specific IgE levels. All correlations were significant, with correlation coefficients and probability values shown in each figure.

Spearman's correlation revealed a significant positive correlation between specific IgE levels of Japanese cedar and alder (*ρ* = 0.571, *P* < 0.001), Japanese cedar and ragweed (*ρ* = 0.540, *P* < 0.001), Japanese cedar and orchard grass (*ρ* = 0.525, *P* < 0.001), alder and ragweed (*ρ* = 0.754, *P* < 0.001), and alder and orchard grass (*ρ* = 0.702, *P* < 0.001). Specific IgE levels for ragweed and orchard grass showed a remarkably strong positive correlation (*ρ* = 0.778, *P* < 0.001) (Figure 2).

### 3.5. Risk Factor Analysis of PFAS Development

Multivariate logistic regression analysis was performed to compare the characteristics of 123 patients with PFAS and 477 patients without PFAS. There was a significant difference between age (OR, 1.12; 95% CI, 1.06–1.19; *P* < 0.001), sAR (OR, 6.93; 95% CI, 1.59–30.34; *P* = 0.010), and alder sensitisation (OR, 6.20; 95% CI, 2.66–14.49; *P* < 0.001) between the two groups with respect to the risk of developing PFAS. Conversely, FA (excluding PFAS) was significant in the group without PFAS (OR, 0.60; 95% CI, 0.38–0.96; *P* = 0.034). Sex, BA, AD, and other-pollen sensitisations were not associated with the risk of developing PFAS (Table 4). Univariate logistic analysis of pollen-specific IgE levels and PFAS development showed that the higher the specific IgE levels of all four pollens, the more likely the patients were to develop PFAS (Table 5).

### 3.6. ROC Analysis between Pollen-Specific IgE Levels and the Development of PFAS

**Figure 3:**
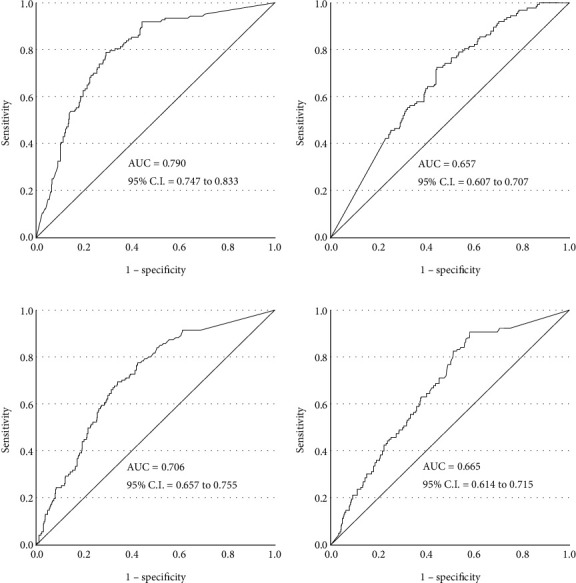
ROC analysis of pollen-specific IgE levels and PFAS development. ROC, receiver operating characteristic; AUC: area under the curve; CI: confidence interval.

The ROC curve was used to calculate the incidence of PFAS by pollen-specific IgE levels (Figure 3). The area under the curve (AUC) of alder and ragweed-specific IgE levels was determined to be of moderate accuracy: 0.790 for alder and 0.706 for ragweed. The cutoff value for alder pollen-specific IgE was 2.54 UA/mL (sensitivity 78.9%, specificity 70.9%), and that for ragweed pollen-specific IgE was 0.89 UA/mL (sensitivity 69.1%, specificity 65.2%). The AUC of Japanese cedar- and orchard grass-specific IgE levels was determined to be of low accuracy: 0.657 for Japanese cedar, and 0.665 for orchard grass.

## 4. Discussion

This is the first study to examine the risk of developing PFAS and association of pollen-specific IgE levels with the development of PFAS in pollen-sensitised Japanese children and adolescents. The prevalence of PFAS was 20.5% among children and adolescents with any pollen sensitisation, 8.5% among 3–6 year-old individuals, 20% among 7–12 year-old individuals, and 36.3% among 13–18 year-old individuals. This is the first report of the prevalence of PFAS in pollen-sensitised preschool children in Japan. The risk factors for the development of PFAS were older age, sAR, and alder sensitisation. Higher pollen-specific IgE levels have been demonstrated to increase the development of PFAS. The OR for the prevalence of PFAS was found to be 36.83 (95% Cl 8.93–151.83, *P* < 0.001) in patients sensitised to Japanese cedar only and those sensitised to Japanese cedar, alder, ragweed, and orchard grass. Alder was most associated with the development of PFAS; the cutoff value for PFAS positivity was 2.54 UA/mL (ROC curve 0.79, sensitivity 78.9%, and specificity 70.9%).

Birch is a major cause of PFAS, although in Japan, its habitat is limited to northern areas, such as Hokkaido. However, alder, which is also a member of the birch family, is abundant in areas south of Honshu. The correlation coefficient between birch and alder is remarkably high in Japan, proving that specific IgE antibodies are formed to alder pollen as well as to birch pollen [26]. Thus, alder-specific IgE was measured in this study. Japanese cedar, ragweed (Asteraceae), and orchard grass (Poaceae) are distributed throughout Japan; these are the primary causes of pollinosis in Japan [19]. Alder-specific IgE levels were evaluated, except Aln g 1, which is more closely related to PFAS because it is related to PR-10. However, testing for Aln g 1 is not covered by insurance in Japan and is, therefore, difficult to examine. Hence, evaluation with alder-specific IgE is more realistic.

As determined by multiple logistic regression, risk factors for the development of PFAS were older age, diagnosis of seasonal allergic rhinitis, and sensitisation to alder. Sex, atopic dermatitis, and bronchial asthma were not risk factors in our study. As in previous reports [16], the prevalence of PFAS increased with age in children and adolescents. There was no difference in the prevalence of allergic rhinitis in G2 and G3; however, there was a significant difference in PFAS. This suggests that the longer the duration of allergic rhinitis, the more likely the patient is to develop PFAS. Previous studies have reported longer duration of allergic rhinitis in patients with PFAS [27, 28]. However, another large study found no significant difference in the duration of birch pollinosis in patients with and without PFAS [29]. In Japan, seasonal allergic rhinitis caused by Japanese cedar, which is less associated with PFAS, precedes other pollens, suggesting that the duration of allergic rhinitis is longer before the onset of PFAS. Asthma is often reported to be a risk factor for developing PFAS [30, 31]; however, our study showed a different result. The strength of the association between pollen sensitisation and asthma may vary by region.

Osterballe et al. reported that sensitisation to birch pollen was associated with a higher rate of PFAS than sensitisation to weed or grass pollen in adults, similar to the results in our study. They also reported that the probability of a clinical reaction to one or more fruits and vegetables was highest (52%) in adults sensitised to all three pollens (birch+grass+mugwort). Moreover, the OR in symptomatic pollen-sensitised adults for a clinical reaction to pollen-related fruits and vegetables was up to four times the OR in adults without pollen symptoms [32]. Our study also demonstrated similar results in children and adolescents. Alder was found to be most associated with PFAS, with an OR of 36.83 among patients sensitised to Japanese cedar only and among those sensitised to Japanese cedar, alder, ragweed, and orchard grass. The risk of developing PFAS increases with the number of pollen sensitisations, proving that Japanese cedar sensitisation is less likely to be associated with PFAS.

Adult patients with Betulaceae allergy and with OAS had higher serum IgE levels to Bet v 1 than patients without OAS [33]. In children, a cutoff value of Bet v 1 was calculated in individuals diagnosed with and without OAS among those sensitised to Bet v 1 and was reported to be 8.2 ISU (ROC curve 0.716, sensitivity 85.7, and specificity 56.8) [34]. In this study, in pollen-sensitised youth aged 3–18 years, the cutoff alder-specific IgE level for developing PFAS was 2.54 UA/mL (AUC 0.79, sensitivity 78.9%, specificity 70.9%). Our study is not comparable because the calculation was based on crude antigen-specific IgE of alder, and the methods for detecting specific IgE levels are different. However, in patients sensitised to Betulaceae pollen, the results are the same: the higher the specific IgE level, the more likely the patient is to develop PFAS. Japanese cedar and orchard grass showed low institutional results with an AUC of <0.7. Ragweed had the next best result after alder with an AUC of 0.706; however, both sensitivity and specificity were low and thus unsatisfactory.

As in previous reports of children with PFAS, the causative agents were predominantly rosaceous fruits, such as peaches, apples, and strawberries, and Cucurbitaceae foods, with melons and watermelons, kiwi, pineapple, and tomato being the most common [14, 16, 17]. Our study showed almost similar results. These foods caused oral symptoms predominantly, most likely due to sensitisation or cross-reactivity to Bet v 1 or profilin.

The prevalence of PFAS in children varies by region and target group. In South Korea, the prevalence of PFAS in children with AD and birch pollinosis aged 2–18 years is 43.5%; in Italy, 24% in children with allergic rhinitis; and in Australia, 4.9% in children aged 4–17 years with atopic disease [14, 35, 36]. In Tokyo, the prevalence of PFAS among 13 year olds with pollen allergy was recently reported to be at 22.9% [17]. About half of the preschool children sensitised to Japanese cedar pollen were also sensitised to either alder, ragweed, or orchard grass. The prevalence of PFAS in our study's population was 8.5%, which was surprisingly higher than expected, although it was limited to infants who were pollen sensitised. The prevalence of PFAS among pollen-sensitised 13–18-year-old individuals in our study group was 36.3%, which was expected to be higher than that in the Tokyo group. The prevalence of non-Japanese cedar pollinosis is reported to be 24% in Tokyo and 31.4% in Tochigi [19]. Therefore, it is possible that the prevalence of PFAS is also higher in our study group than in the Tokyo group due to the large number of patients with alder, ragweed, and orchard grass pollinosis in our group. Since most pollen allergies in Japan are caused by Japanese cedar pollen, we expected the prevalence of PFAS among allergic rhinitis patients or pollen sensitised patients to be lower than that in other countries; however, 74% of Japanese cedar sensitised patients were also sensitised to either alder, ragweed, or orchard grass. Thus, the result would not have been lower than in other countries. In addition, Yamamoto-Hanada et al. [37] reported an increase in PR-10-related components among individuals aged 5–9 years, and in our study, the prevalence of PFAS increased from G1 to G2, suggesting a sharp increase in sensitisation related to PR-10 in the first half of elementary school.

This study demonstrated that higher specific IgE levels in four pollens (alder, Japanese cedar, ragweed, and orchard grass) are associated with a higher risk of developing PFAS. Although Japanese cedar pollen is considered to have a low association with PFAS, high Japanese cedar pollen-specific IgE levels were also a risk factor in this study. This may be due to the fact that in our study, each pollen-specific IgE level was correlated with each other and, thus, high specific IgE levels of other-pollens were also associated with high levels of Japanese cedar pollen.

The results of this study highlight the need to prevent multispecies pollen sensitisation and elevation of pollen-specific IgE levels, particularly of alder. Thus, efforts should be made to limit pollen exposure, subsequently preventing atopic dermatitis and allergen sensitisation from infancy from the perspective of the allergic march.

This study had some limitations. First, PFAS was defined by clinical history based on questionnaires and pollen sensitisation. Food-specific IgE levels and skin prick tests were not performed. However, oral food challenge tests and specific IgE tests may not guarantee a true PFAS due to the antigenic instability of commercial antigens. Rather, a careful clinical history may be sufficient to diagnose PFAS. Anhoej et al. [38] reported that the clinical history of OAS caused by apples had negative and positive predictive values of 100% and 92%, respectively, compared with OFC. Skypala et al. [39] found that their questionnaire-based diagnosis had negative and positive predictive values of 91% and 92%, respectively, compared with a protocol that combined a history, oral load test, and skin prick test performed by experts. In this study, the diagnosis of PFAS was reliable because allergy specialists took a clinical history and only systemic symptoms (itching or urticaria, cough, dyspnea, vomiting, and/or anaphylaxis) were excluded in this study as they are considered as Class 1 allergies.

Second, selection bias is possible because the evaluation was conducted at a single university hospital. Therefore, it is possible that many severely allergic patients were included.

Third, it is more useful to test for the allergic component of each pollen or plant; however, such tests are not covered by insurance and not all patients can be tested. Therefore, pollen-specific IgE levels are useful for predicting the development of PFAS in clinical practice in the future.

Fourth, pollen-specific IgE levels and PFAS symptoms have been influenced by seasons. However, as the seasons were not standardised in this study, there may have been variations in pollen-specific IgE levels and PFAS symptoms. Nevertheless, we believe that the large sample size is sufficient to cover this bias.

## 5. Conclusions

Preschool children develop PFAS, and the prevalence was 8.5% in those with any pollen sensitisation. Risk factors of PFAS were older age, diagnosis of allergic rhinitis, alder sensitisation, higher pollen-specific IgE levels, and increased number of pollen sensitisations in this study group of Japanese children and adolescents with any pollen sensitisation. Alder-specific IgE levels can predict the development of PFAS.

## Figures and Tables

**Table 1:** Clinical characteristics of the participants.

	Total	Age, in years			*P* value 1 (for all group)	*P* value 2		
		3–6 (G1)	7–12 (G2)	13–18 (G3)		G1 vs. 2	G1 vs. 3	G2 vs. 3
*N*	600	199	244	157				
Sex (male : female)	378 : 222	124 : 75	158 : 86	96 : 61	0.743	—	—	—
Age (median : min–max)	9 : 3-18							
Allergic diagnosis								
Food allergy^†^	365 (60.8%)	141 (70.9%)	149 (61.1%)	75 (47.8%)	**<0.001**	**0.035**	**<0.001**	**0.023**
Atopic dermatitis	288 (48.0%)	105 (52.8%)	113 (46.3%)	70 (44.6%)	0.244	—	—	—
Bronchial asthma	200 (33.3%)	57 (28.6%)	90 (36.9%)	53 (33.8%)	0.186	—	—	—
Seasonal allergic rhinitis	462 (77.0%)	110 (55.2%)	210 (86.1%)	142 (90.4%)	**<0.001**	**<0.001**	**<0.001**	0.522
PFAS	123 (20.5%)	17 (8.5%)	49 (20.0%)	57 (36.3%)	**<0.001**	**<0.001**	**<0.001**	**<0.001**
Sex (male : female)	75 : 48	13 : 4	28 : 21	34 : 23	0.602	0.296 (G1)^‡^	0.243 (G2)^‡^	0.865 (G3)^‡^
Pollen-specific IgE-positive rate								
Alder	371 (61.8%)	95 (47.7%)	165 (67.6%)	111 (70.7%)	**<0.001**	**<0.001**	**<0.001**	0.581
Japanese cedar	594 (99.0%)	195 (98.0%)	244 (100%)	155 (98.7%)	0.099	—	—	—
Ragweed	338 (56.3%)	87 (43.7%)	150 (61.4%)	101 (64.3%)	**<0.001**	**<0.001**	**<0.001**	0.598
Orchard grass	380 (63.3%)	91 (45.7%)	167 (68.4%)	122 (77.7%)	**<0.001**	**<0.001**	**<0.001**	0.052

^†^Excluding PFAS. *P* value 1: Fisher's exact test. *P* value 2: pairwise comparison (Fisher's exact test with Bonferroni correction), ^‡^*P* value 2: comparison within age groups for gender (Fisher's exact test).

**Table 2:** Causative foods of PFAS.

Allergen	Total
Total (*N*)	123
Peach	49
Apple	31
Kiwi	30
Melon	26
Pineapple	19
Strawberry	19
Tomato	17
Pear	16
Watermelon	14
Orange	13
Cherry	13
Banana	12
Cucumber	9
Mango	5
Eggplant	6
Avocado	4
Loquat	4
Grape	3
Plum	2
Carrot	1
Celery	1

**Table 3:** Chi-square test of PFAS development with the Japanese cedar only group versus the multiple pollen sensitisation group.

	OR	95% CI	*P* value
Japanese cedar only vs. two antigens	9.74	2.08-45.57	<0.001
Japanese cedar only vs. three antigens	21.28	4.67-96.88	<0.001
Japanese cedar only vs. four antigens	36.83	8.93-151.83	<0.001

OR: odds ratio; 95% CI: 95% confidence interval.

**Table 4:** Multivariate logistic regression analysis for the development of PFAS.

	PFAS	No PFAS	Reference	OR	95% CI	*P* value
N	123	477				
Age, years	12 (4-18)	8 (3-18)	Per 1 year	1.12	1.06–1.19	**<0.001**
Male	75 (61.0%)	303 (63.5%)	vs. female	0.67	0.42–1.06	0.087
Female	48 (39.0%)	174 (36.5%)	vs. male	1.49	0.94–2.38	0.087
Allergic diagnosis						
Food allergy^†^	66 (53.7%)	301 (63.1%)	vs. negative	0.60	0.38–0.96	**0.034**
Atopic dermatitis	76 (61.8%)	247 (51.8%)	vs. negative	1.20	0.76–1.89	0.439
Bronchial asthma	32 (26.0%)	168 (35.2%)	vs. negative	0.63	0.38–1.03	0.064
Seasonal allergic rhinitis	121 (98.4%)	340 (71.3%)	vs. negative	6.93	1.59–30.34	**0.010**
Pollen-specific IgE-positive rate (%)						
Alder	114 (92.7%)	257 (53.9%)	vs. negative	6.20	2.66–14.49	**<0.001**
Japanese cedar	123 (100%)	471 (98.7%)	vs. negative	—	—	—
Ragweed	100 (81.3%)	238 (49.9%)	vs. negative	1.20	0.62–2.33	0.585
Orchard grass	107 (87.0%)	273 (57.2%)	vs. negative	1.08	0.51–2.30	0.841

OR: odds ratio; 95% CI: 95% confidence interval. Subjects: all patients (*N* = 600). ^†^Excluding PFAS.

**Table 5:** Univariate logistic regression analysis for the development of PFAS.

	PFAS	No PFAS	Reference	OR	95% CI	*P* value
N	123	477				
Pollen-specific IgE levels (median: min–max)						
Alder	16.9: <0.1–100	0.48: <0.1–100	Per 1 (LN [UA/mL])	1.024	1.017–1.030	**<0.001**
Japanese cedar	78.9: 1.64–100	34.4: <0.1–100	Per 1 (LN [UA/mL])	1.013	1.008–1.019	**<0.001**
Ragweed	2.25: <0.1–100	0.34: <0.1–100	Per 1 (LN [UA/mL])	1.020	1.007–1.035	**<0.001**
Orchard grass	2.67: <0.1–100	0.63: <0.1–100	Per 1 (LN [UA/mL])	1.012	1.004–1.020	**<0.001**

OR: odds ratio; 95% CI: 95% confidence interval. Subjects: all patients (*N* = 600).

## Data Availability

The data that support the findings of this study are available on request from the corresponding author. The data are not publicly available due to privacy reasons.
